# Diagnosis of early-onset sarcoidosis with non–classical symptoms

**DOI:** 10.1186/1546-0096-12-S1-P269

**Published:** 2014-09-17

**Authors:** Zübeyde Gündüz, Ayşenur Paç Kısaarslan, Ruhan Düşünsel, Afig Berdeli, Betül Sözeri, Hakan Poyrazoğlu

**Affiliations:** 1 Pediatric Rheumatology, Erciyes University faculty of medicine, Kayseri, Turkey; 2 Molecular Genetic, Ege University Molecular Genetic, İzmir, Turkey

## Results

A 15-month-old male patient was referred to pediatric rheumatology for evaluation of non-pruritic skin eruption most prominent on arm and leg, fever and lymphadenopathy. These symptoms began when the patient was four months old. Several antibiotics therapies had been examined. The maximal convelescence period was a month. There were no consanguinity and family history. Physical examination showed generalized, maculopapulary eruption especially on arm and leg, lymph node on left cervical and axillary region, and persistant fever. In laboratory examinations, acute phase reactans were found to be high. The serology of EBV, CMV, toxoplasma, brucella, tularemia, bartonella henselae, and Quantiferron test results were negative. Immunologic evaluations contained hemogram, blood smear, immunoglobulins, fagotest, and CD panel were normal. Urine calcium/creatinin ratio, serum ACE levels were normal. A skin biopsy taken from a papule showed subepidermal non- langerhas histiocytes and a non-caseating granuloma. A lymph node biopsy showed granulomatous inflammation. Mediastinal lymph node was absent on thorax CT evaluation. His ophtalmological examination was normal. The granulomatous autoinflammatory disease was diagnosed. Prednisolone 2 mg/kg was administered which reduced symptoms and patient’s complaints. Then, the steroid dose was dropped. The fever and rash were absent, but the acute phase reactans were still high. His blood samples were referred for genetic testing NOD2 mutations. Methotrexate was added to the treatment, but the flair was occured two mounths later. NOD2 mutations was detected to be P268S heterozigot and V955I heterozigot. Since the patient’s response to anakinra was poor, TNF bloker was later added to the treatment.

## Conclusion

Granulomatous autoinflammatory diseases include Blau syndrome and early-onset sarcoidosis, both are caused by mutations in the NOD2/CARD15 gene, inherited autosomal dominant and sporodic form of disease, respectively. Clinical triads are üveitis, arthritis and granulomatous dermatitis whereas the symptops we observed were fever, granulomatous dermatitis, and lymphadenopathy.

## Disclosure of interest

None declared.

